# Myocardial iron assessment by T2* cardiovascular magnetic resonance at 3 Tesla

**DOI:** 10.1186/1532-429X-16-S1-P310

**Published:** 2014-01-16

**Authors:** Mohammed H Alam, Gillian C Smith, Taigang He, John Paul Carpenter, Arun J Baksi, Ricardo Wage, Peter Drivas, Karen Symmonds, David Firmin, Dudley J Pennell

**Affiliations:** 1 NIHR Cardiovascular Biomedical Research Unit, Royal Brompton Hospital, London, UK; 2 Imperial College London, London, UK

## Background

Chronic iron overload is prevalent and prognosis relates to the severity of cardiac siderosis. The black blood (BB) T2* technique is validated, with calibration against myocardial tissue iron at 1.5T. There are potential advantages of 3T over 1.5T in assessing T2*, but preliminary studies at 3T used white blood imaging, had small patient numbers, methodological differences, and poor signal-noise ratio in severe iron overload precluding assessment across a wide dynamic range. A novel noise correction (NC) algorithm is superior to other techniques in severe iron loading. We assessed BB T2* iron measurement at 3T against the 1.5T gold standard and incorporated the NC algorithm.

## Methods

120 subjects (65 male, aged 14 to 81 years) were recruited, comprising 20 controls and 100 patients (thalassemia major 43, sickle cell disease 15, haemochromatosis 11, other iron overload conditions 31). BB T2* images at 1.5T (Avanto, Siemens, Erlangen, Germany), and 3T (Skyra) were acquired in the short axis. 20 patients had repeat studies on the same day for reproducibility. Septal regions of interest were analyzed using CMRtools and the novel NC algorithm was applied only if T2* curve fitting was unsatisfactory after truncation. All patient images were ranked for artefact severity on an ordinal scale from 0 (uninterpretable images) to 5 (no septal artefact).

## Results

**Figure 1:**
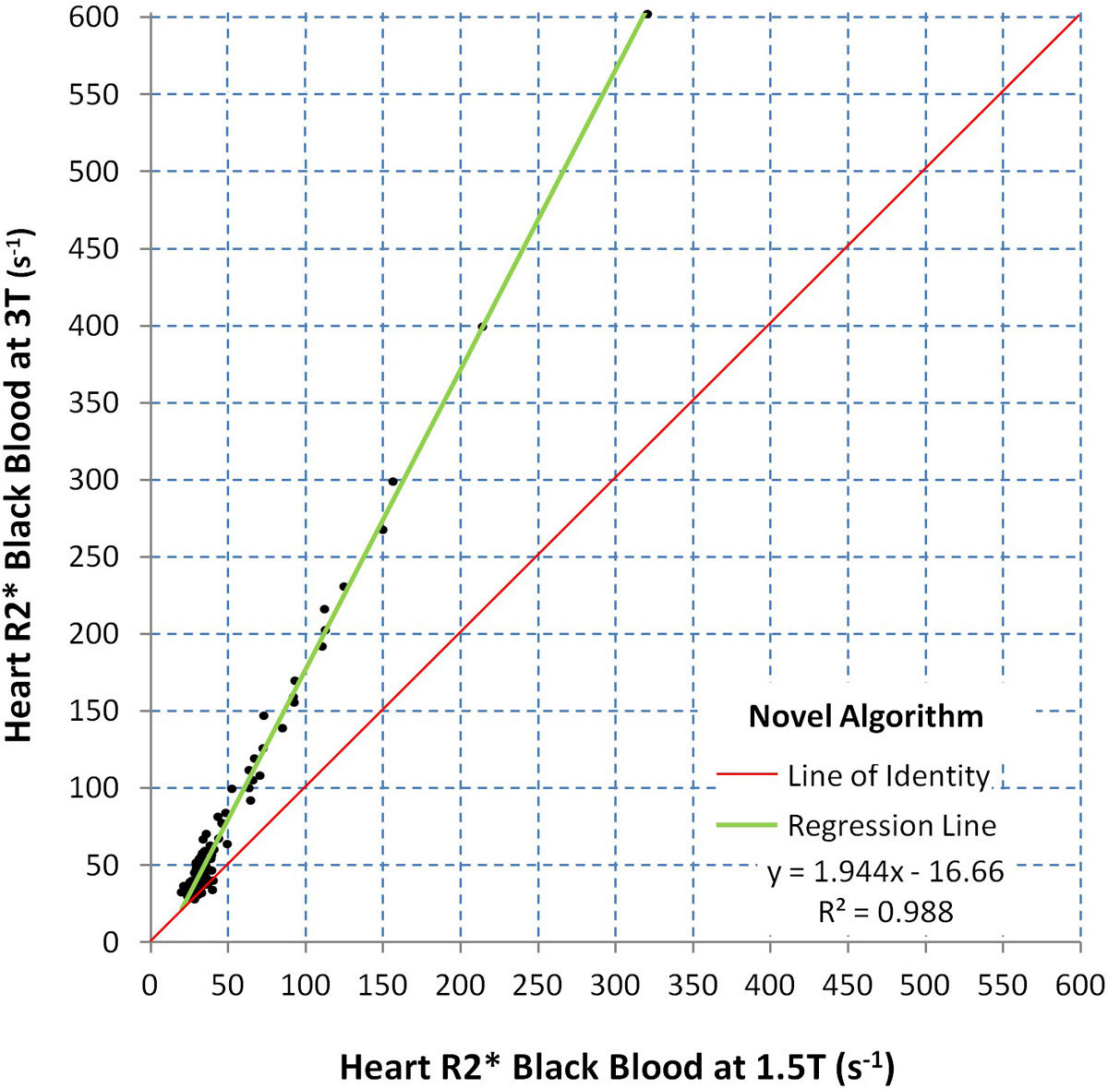
Scatter plot showing the relationship between black blood myocardial T2* at 3T with 1.5T after application of the novel noise correction (NC) algorithm.

Median BB T2* at 1.5T was 29.8 ms (range 3.12-50.5 ms) in patients and 30.7 ms (range 22.6-40.4 ms) in controls. Respective values at 3T were 21.4 ms (range 1.70-35.5 ms) and 24.9 ms (range 13.2-29.4 ms). There was a strong linear correlation between BB R2* (1000/T2*) at 3T and 1.5T (slope 1.94, intercept -16.7, R-squared = 0.988; Figure 1). There was good intra-observer, inter-observer and inter-study reproducibility of BB T2* measurement at 3T with coefficients of variation (CoV) of 3.1%, 4.0% and 6.5%, although these were inferior to 1.5T CoV, which were 1.6%, 2.8% and 4.4% respectively. Mean artefact score at 3T was significantly inferior to 1.5T (4.11 vs 4.39; p = 0.001).

## Conclusions

In this large patient group, cardiac T2* at 3T was feasible and reproducible using BB T2* and the novel NC algorithm which allowed incorporation of the severe iron overload data for successful calibration against the 1.5T BB T2* gold standard. The T2* at 3T was approximately half that at 1.5T. Further validation of this 3T method may improve access to non-invasive cardiac iron measurement.

## Funding

This research was supported by the NIHR Cardiovascular Biomedical Research Unit at Royal Brompton & Harefield NHS Foundation Trust and Imperial College London.

